# Cutaneous malignancies in patients with Parkinson’s disease at a dermato-oncological university centre in Hungary

**DOI:** 10.3389/fonc.2023.1142170

**Published:** 2023-05-19

**Authors:** Veronika Tóth, Stefani Christina Diakoumakou, Enikő Kuroli, Béla Tóth, Daniella Kuzmanovszki, József Szakonyi, Kende Kálmán Lőrincz, Beáta Somlai, Sarolta Kárpáti, Péter Holló

**Affiliations:** ^1^ Department of Dermatology, Venereology and Dermatooncology, Semmelweis University, Budapest, Hungary; ^2^ General Hospital Sismanoglio-Amalia Fleming, Athens, Greece

**Keywords:** Parkinson’s disease, risk, skin malignancy, melanoma, nonmelanoma skin cancer

## Abstract

**Background:**

The possible correlation between melanoma and Parkinson’s disease (PD) has been intensively studied. In this work, we aimed to assess the coincidence of skin malignancies and PD at a dermato-oncological university centre in Central-Eastern Europe, Hungary.

**Methods:**

From 2004 to 2017, a retrospective analysis of the centre’s database was performed based on International Statistical Classification of Diseases-10 codes.

**Results:**

Out of the patients who visited the clinic during the study period, 20,658 were treated for malignant skin tumours. Over the 14 years, 205 dermatological patients had PD simultaneously, 111 (54%) of whom had at least one type of skin malignancy: melanoma (n=22), basal cell carcinoma (BCC) (n=82), or squamous cell carcinoma (SCC) (n=36) (in some patients, multiple skin tumours were identified). Compared to the age- and sex-matched control group, patients with PD had a significantly lower risk for basal cell carcinoma (OR, 0.65; 95% CI, 0.47–0.89, p=0.0076) and for all skin tumours (OR, 0.74; 95% CI, 0.56–0.98, p=0.0392) but not for melanoma.

**Conclusions:**

We found a decreased risk of all skin tumours and basal cell carcinoma and an unchanged risk of melanoma among patients with PD. However, it should be kept in mind that some large-scale meta-analyses suggest a higher incidence of melanoma after a diagnosis of PD, indicating the importance of skin examination in this vulnerable population.

## 1 Introduction

### 1.1 Parkinson’s disease and melanoma

The connection between Parkinson’s disease (PD) and melanoma was first described by Skibba in 1972, presenting a case of multiple primary melanomas in a patient with PD treated with levodopa (1). Afterwards, although levodopa remained a “gold standard” in the treatment of PD, several reports presented melanoma cases among patients treated with levodopa (2–7). These observations resulted in concern with levodopa treatment, and in some countries, the warning of the possible risk was included in the drug leaflet. A case of melanocytic hyperactivation simulating acral lentiginous melanoma in a patient with PD treated with levodopa was published recently (8). The theory suggests that the association between levodopa therapy and melanoma development is based on the shared biochemical pathway of dopamine and melanin synthesis, yet currently, most reports deny this supposed connection (9–15).

The mechanism by which melanoma develops more frequently in PD patients and why patients with melanoma more frequently have PD than the general population have not yet been clarified. Although several mechanisms have been proposed, the data are contradictory. The background of both diseases seems to be multifactorial, and both genetic and environmental factors may play a role in their pathomechanisms (8, 9, 16–24).

Mutations in shared genetic factors such as *Parkin*, *DJ-1*, *LRRK2*, *α-synuclein*, *PLA2G6*, *EIF4G1*, and *SYNJ1* gene/protein can contribute to the association between the two diseases. *Parkin* is a tumour suppressor gene that encodes a ubiquitin ligase found in several cancers and up to half of patients with early-onset familial PD. Loss or mutation of the gene interferes with the degradation of cyclin E; consequently, its level increases and forces the cell to divide faster (25, 26). The role of the mutant *DJ-1* oncogene, a sensor of oxidative stress, was found in different tumour types and proven to cause early-onset hereditary PD. Mutant *LRRK2* causes both familial and sporadic PD and is responsible for 30%–40% of late-onset PD cases in the Ashkenazi Jewish population and North African Arab Berbers population. A multinational study from five centres demonstrated that *LRRK2 G2019S* mutation carriers have an overall increased risk, especially for breast and hormone-related cancers, compared to non-carriers (27). Further theory has been based on alpha-synuclein, a small soluble protein expressed primarily in presynaptic terminals of neural tissue and in both benign and malignant melanocytic lesions and certain other tumours. Alpha-synuclein production is equally increased in dopaminergic neuronal cells in patients with PD and in melanoma cells (28–30). Pigmentation can be another potential common pathway; according to a meta-analysis, red hair and certain *MC1R* gene variants, which are both risk factors for melanoma, can increase the occurrence of PD as well (31). In addition, changes in low-penetrance genes (e.g., *glutathione S-transferase M1 gene*, *cytochrome p450 debrisoquine hydroxylase*, and *vitamin D receptor gene*) together with environmental factors (e.g., socioeconomic status, smoking, and sun-bathing habits) can equally contribute to the association between the two diseases (32, 33). However, certain authors deny any association or the role of common genetic background (34, 35).

Although the epidemiological data are occasionally controversial, large-scale meta-analyses mostly confirmed the positive association between PD and melanoma. The authors of a large meta-analysis involving 40 studies (2,317,408 PD patients) found that PD patients had decreased risks for urogenital, gastrointestinal, lung, and haematological malignancies but increased risks for melanoma and brain cancer compared to controls (36). Similarly, in another meta-analysis of 14 studies, PD patients had decreased risks for general, colon, rectal, and lung cancer but increased risks for brain cancer and melanoma. In contrast, when the *LRRK2 G2019S* mutation was present in PD patients, increased risks for general, colon, haematological, and brain cancer were observed (37). A meta-analysis of the cancer incidence of 577,013 patients suffering from eight different central nervous system disorders showed that the co-occurrence of PD and melanoma was higher, while general, lung, prostate, and colorectal cancer co-occurrence was lower in cases of PD than in control patients (38). The results of a recent systematic review and meta-analysis of 292,275 PD patients in 24 studies showed that the risk of melanoma after, but not before, the diagnosis of PD was significantly higher (39). Another meta-analysis also confirmed the importance of the time of melanoma diagnosis; the authors found that the occurrence of melanoma was significantly higher only after the diagnosis of PD (40).

### 1.2 PD, non-melanoma skin cancers, and extracutaneous tumours

The connection between PD and non-melanoma skin cancer (NMSC) is contradictory. Some authors have reported that patients suffering from PD have a higher risk for NMSCs (16, 18, 41), especially for basal (42, 43) and squamous cell carcinoma of the head and neck region (44, 45). Others described a significantly lower frequency of any skin cancer and non-melanoma skin cancers (37, 46). Lerman et al. found weak or no association between PD and squamous cell carcinoma (47). PD can be associated not only with skin malignancies but also with a wide spectrum of skin disorders, such as seborrheic dermatitis, sweating disorders, bullous pemphigoid, rosacea, and perioral dermatitis (48, 49).

Various studies have investigated the connection between PD and extracutaneous malignancies. Many authors found a significantly lower risk for all malignancies among patients with PD, including colorectal, lung, and larynx cancer, while breast, prostate, renal, and thyroid cancer risk was reported to be higher than expected (16, 18, 38, 50, 51). In contrast, Lin et al. found that, in Taiwan, PD was not associated with breast, ovarian, or thyroid cancers, but increased hazard ratios were found in cases of 16 cancers, including gastrointestinal tract cancers and lung and brain tumours (52). Bajaj et al. conducted a large review of the literature involving more than 100,000 patients. They found significantly reduced overall cancer risk ratios among patients with PD (53).

Since studies focusing on the association of PD and skin tumours were mainly carried out in the USA (15, 19, 20, 54–58) and in Western European populations, such as the United Kingdom (50, 59), Germany (38), Sweden (60), Denmark (13, 38, 61, 62), Portugal (49), Israel (10, 63), and Taiwan (50, 52), we aimed to assess the co-occurrence of skin malignancies and PD among patients from an academic dermato-oncology centre in Hungary, a Central-Eastern European country.

## 2 Materials and methods

### 2.1 Patients

A retrospective analysis of the database of the Department of Dermatology, Venereology, and Dermatooncology at Semmelweis University was performed between 1 January 2004 and 31 December 2017. The total number of patients who visited the department during the 14-year examination because of any skin malignancy was 20,658. To identify patients with melanoma, NMSC, and PD, we used International Statistical Classification of Diseases-10 (ICD-10) codes; patients with melanoma were selected by the c4300–c4390 codes, squamous cell carcinoma by the c4401–c4491 codes, and basal cell carcinoma by the c4402–c4492 codes. The patients with PD were collected based on the ICD-10 code G20. Combining the patient populations of the four different diagnoses, we selected those patients who had melanoma or NMSC associated with PD. Data on skin tumours and PD were obtained by reviewing the patient’s medical history.

*Control group*. Because the mean age of the patients diagnosed with PD and skin tumours was different, in addition to these diseases affecting the two sexes differently, for each patient with PD, five age- and sex-matched controls without PD were selected randomly from the patients of the department from 2004 to 2017. These five control patients had the same sex and were born in the same year as the studied patient. Consequently, 205 patients with PD associated with skin disease were matched with 1,025 age- and sex-matched control patients with skin disease but no PD.

### 2.2 Statistical analysis

First, descriptive statistics were performed. The association between PD and melanoma and basal and squamous cell carcinoma was assessed separately. To examine the odds of having skin malignancy in PD patients, we used GraphPad Prism 9.3.1 (147) software. Odds ratios (ORs) and their 95% confidence intervals (CIs) were calculated for each group from contingency tables. An OR >1 indicated a positive association, and an OR <1 indicated a negative association between a given skin tumour and PD. Comparing the ORs of different tumour types, we used the case numbers (number of skin tumours) instead of the number of patients because, contrary to most other malignant disorders, tumours of the skin are often multiple. Consequently, in the case of calculation with the number of patients instead of the number of skin neoplasms, cases were lost. Since we had a small sample size, to calculate the correlation between our categorical variables, we used Fisher’s exact tests to determine whether the difference was statistically significant. A probability value of <0.05 was considered statistically significant.

## 3 Results

### 3.1 Types of skin malignancies

From 2004 to 2017 at the Department of Dermatology, Venereology, and Dermatooncology at Semmelweis University, 20,658 patients were treated for skin malignancy. A total of 6,300 patients had melanoma, 11,983 had basal cell carcinoma, and 2,375 had squamous cell carcinoma.

#### 3.1.1 PD group

Over the 14 years, 205 patients visited the department due to a skin disease and PD simultaneously. The ratio of male to female patients was 61.5% to 38.5%. Of the 205 patients, 94 had no skin malignancy, while 111 (54%) patients had malignant skin tumours. The mean age of the studied patients was 78 ± 8.4 years, with a median age of 79 years. The youngest patient was 47 years old, and the oldest patient was 96 years old. The mean age at PD onset was 72.2 ± 9.5 years, while the mean age at the diagnosis of the first skin malignancy at the department was 77.9 ± 8 years (**Table 1**).

**Table 1:** Demographic data of patients with simultaneous malignant or non-malignant skin disease and PD.

	All	Male	Female
All PD + skin malignancy PD without skin malignancy	205 111 94	126 (61.5%) 72 54	79 (38.5%) 39 40
Mean age± SD Median age Mean age at PD onset Median age at PD onset Mean age at the diagnosis of skin tumour Median age at the diagnosis of skin tumour	78 ± 8.4 79 72.2 ± 9.5 75 77.9 ± 8.4 79	78.5 ± 7.8 80 73.3 ± 9.3 74 78.2 ± 7.3 79.5	75.5 ± 14 77 73 ± 9.8 73 77.4 ± 10.1 78

PD, Parkinson’s disease; SD, standard deviation.

Multiple skin tumours were frequent among these 111 patients; 65 of them had only one skin malignancy, and in the remaining 46 patients, multiple tumours occurred. Altogether, 140 skin malignancies (positive events), 22 melanomas, 82 basal cell carcinomas, and 36 squamous cell carcinomas were diagnosed in the 111 patients. In addition, we found some rare skin tumours simultaneously with PD: two trichilemmal carcinomas, a malignant fibrous histiocytoma, an atypical fibroxanthoma, and a Merkel cell carcinoma. There were 27 patients who had different types of skin malignancies simultaneously, and one patient had 35 malignant skin tumours, including melanomas, trichilemmal carcinomas, and basal and squamous cell carcinomas.

#### 3.1.2 Control group

We randomly coupled each of the 205 patients with PD with five age- and sex-matched patients who did not have PD from the department from 2004 to 2017. Consequently, 205 patients with PD and 1,025 control patients were matched. A total of 560 patients were negative for skin tumours, while 465 patients had a total of 1,127 skin malignancies (total events): 132 melanomas, 752 basal cell carcinomas, and 243 squamous cell carcinomas (on some patients, multiple skin tumours occurred).

### 3.2 Risk of skin malignancy

Assessing the odds of all skin malignancies, it proved to be significantly decreased among the patients with PD (OR, 0.74; 95% CI, 0.56–0.98; p=0.0392). The odds proved to be significantly decreased as well in cases of basal cell carcinoma and PD (OR, 0.65; 95% CI, 0.47–0.89; p=0.0076). On the other hand, the results did not show a significant correlation between PD and melanoma or PD and squamous cell carcinoma (**Table 2**).

**Table 2:** Comparison of skin malignancies in patients with or without PD; odds ratios of skin malignancies among patients with PD.

	Patients with PD (positive events)	Patients without PD (total events)	OR	95% CI	p
Number of patients without any malignant skin tumour	94	560			
Number of patients with any malignant skin tumour	111	465	1.42	1.05–1.91	0.026
Number of all malignant skin tumours	140	1127	0.74	0.56–0.98	0.0392
Number of melanomas	22	132	0.99	0.61–1.64	> 0.9999
Number of BCCs	82	752	0.65	0.47–0.89	0.0076
Number of SCCs	36	243	0.88	0.59–1.34	0.6062

BCC, basal cell carcinoma; SCC, squamous cell carcinoma; PD, Parkinson’s disease; OR, odds ratio; CI, confidence interval.

p-values were calculated by Fisher’s exact test.

### 3.3 Sex differences in the distribution of skin malignancies among PD patients

Single and multiple skin malignancies equally developed more frequently in men than in women, which was noticeable for every type of skin tumour. Seven of 19 patients suffering from melanoma had additional basal cell carcinomas, and two had additional squamous cell carcinomas as well (**Table 3**).

**Table 3:** Sex distribution of skin malignancies in patients with Parkinson’s disease.

	All	Male	Female
Number of all skin tumours	140	99	41
Number of melanomas	22	16 (3 multiple)	6
Number of BCCs	82	56 (33 multiple)	26 (7 multiple)
Number of SCCs	36	27 (13 multiple)	9 (2 multiple)

BCC, basal cell carcinoma; SCC, squamous cell carcinoma.

The average age at melanoma diagnosis was 73±11.5 years (men, 75.5±6.8 years; women, 69±16 years). The most frequent localisation was the head–neck area (41% of the melanomas). All of these patients were treated orally with anti-Parkinson’s agents. A total of 10 patients received levodopa/benserazide, six received amantadine, five received ropinirole, five received selegiline, three received rasagiline, two received levadopa/carbidopa/entacapone, and two received pramipexole. One patient was treated with rotigotine, biperiden hydrochloride, procyclidine, and carbidopa/levodopa.

The average Breslow thickness of all melanomas among male patients was 8.27±10.9 mm. Out of the 13 male patients with melanoma, six had advanced, thick tumours with a high average thickness of 12.6±12.5 mm. Two of these six patients also developed second primary *in situ* melanoma (n=3). We had a male patient who had 35 different malignant skin tumours (one melanoma, nine basal cell carcinomas, 24 squamous cell carcinomas, and one trichilemmal carcinoma), most of which developed in sun-exposed areas. The patient’s PD was diagnosed 8 years earlier than the melanoma, and he was treated with amantadine and levodopa/carbidopa/entacapone followed by levodopa/benserazide.

In two of the six female patients, the histological data of the primary melanoma remained unknown. The other four melanomas were thin and belonged to the IA or IB tumour stage.

## 4 Discussion

During the 14 years examined, we found a significant association between PD and skin malignancies, with lower odds of having all skin malignancies or basal cell carcinoma among the PD patients of our department.

Some data suggest that the total cancer risk is reduced in patients with PD (51, 62, 64). In the largest recent meta-analysis according to data from more than 17,000,000 patients in 63 studies, PD was inversely associated with total cancer risk, while there was no significant association between NMSC and PD (65). Reviewing the literature, Bajaj et al. established a decreased risk of non-melanoma skin cancer in cases of PD (OR, 0.79) (52). As in the case of extracutaneous tumours, many authors found a decreased risk of basal cell carcinoma, while others established a positive connection (40, 48, 49, 66). These data are partially in accordance with our results showing decreased odds for all skin cancers and basal cell carcinoma. Since BCC is the most frequent human malignancy, its lower risk can be in the background of the decreased total skin tumour risk in cases of PD.

Among the patients with PD, the number of basal cell carcinomas was less than half (25 vs. 57), and the number of squamous cell carcinomas was approximately one-third (10 vs. 26) in women than in men. A higher number of basal and squamous cell carcinomas was also reported in men in the general population, where the risk factors for multiple NMSCs are age, history of ≥10 sunburns, male sex, and family history of melanoma (67–69). Among our patients, multiple NMSCs were four times more common in men than in women. Moreover, we had a male patient who had 35 primary skin malignancies. This number of NMSCs associated with PD is unique in the literature (44, 45). Salemi et al. reported a 79-year-old male and a 98-year-old female patient who had PD and multiple squamous cell carcinomas in the head–neck region. They supposed that a proapoptotic gene (*LDOC1L, leucine zipper, downregulated in cancer 1-like gene*) may have played a pathogenetic role in the development of these malignancies (46). Our patient worked as a physical education teacher, which suggests periodic outdoor activities. He took amantadine, a light-sensitising drug against PD. While most of his malignancies developed in sun-affected areas, UV radiation and drug-induced photosensitivity may have played a role in tumour formation.

Our data showed no association between PD and squamous cell carcinoma or melanoma, which contradicts the results of some large-scale meta-analyses (36–38).

Some explanations for this contradiction may be raised. For example, Lee et al. did not find increased melanoma risk in their meta-analysis among *LRRK2 G2019S* gene mutation carrier PD patients (37). Genetic analysis was not part of the study, and the authors could not determine whether it affected the results. Another reason for this contradiction might be technical: our study was performed at a dermato-oncological university centre in Central-Eastern Europe. The medical history of each patient treated for skin cancer was documented thoroughly. To identify the PD patients, we used the ICD-10 codes. However, if the neurodegenerative disease was mentioned in the medical history but not represented with an ICD-10 code, we failed to find the patient, who consequently dropped out of the study. Furthermore, most of the large reviews were conducted in Western Europe and the USA (37, 39, 40). It may also be hypothesised that in these countries, the healthcare system pays more attention to and cares for elderly and lonely PD patients, while in Central-Eastern Europe, these patients do not reach dermato-oncological centres in time with a skin tumour, which may result in a lower number of patients in these centres suffering from both melanoma and PD.

The sex distribution of our patients was in accordance with the literature that reports a higher incidence rate of PD among men than women (70). In the group of male patients treated for PD and melanoma, the average Breslow thickness was high (8.27±10.9 mm). All four female patients with known primary tumours had early-stage melanomas, with an average Breslow thickness of 0.5±0.5 mm. The thicker tumours of our male patients treated with PD are in accordance with the reported thicker melanomas among men in the general population (71, 72). The most frequent localisations of melanomas were the sun-exposed skin surfaces, particularly the head–neck area and the arms (68.5%), which emphasises the role of UV radiation and anti-Parkinson’s drug-induced photosensitivity as two potential environmental factors (30, 44–47). Levodopa was the most often administered anti-Parkinson’s agent in our patients with concomitant melanoma (13/19). This drug is the gold standard in the treatment of PD, although it was previously considered a predisposing factor for melanoma; currently, most authors deny the connection (9–15). Since Breslow thickness is one of the most important independent prognostic factors for melanoma (73), the high rate of thick tumours mostly developed on sun-exposed skin surfaces and the non-decreased risk of melanoma emphasise the importance of UV protection and regular oncodermatological screening among men living with PD.

In conclusion, this is the first study that analysed the association between skin tumours and PD in Central-Eastern Europe. While the majority of the studies focused on the risk of melanoma in PD patients, our examination was extended to other skin malignancies, such as basal and squamous cell carcinoma. The risk of all skin malignancies was significantly lower, which may be related to the similarly decreased risk of basal cell carcinoma in patients with PD. Despite this encouraging result, the OR of melanoma was not influenced. Moreover, we noticed a large proportion of high-risk melanomas among men, mostly in sun-exposed areas, while women had incipient melanomas. In conclusion, UV protection and regular oncodermatological screening remain highly important, especially for male patients treated for PD, to prevent or to notice potentially lethal melanoma tumours earlier.

## Data availability statement

The original contributions presented in the study are included in the article/supplementary material. Further inquiries can be directed to the corresponding author.

## Ethics statement

The study plan was reviewed and approved by Semmelweis University Regional and Institutional Committee of Science and Research Ethics. Approval number: 209/2021.

## Author contributions

Conceptualization: BS and VT; methodology: BT; investigation: DK; software: KL and JS; original draft preparation: SD; review and editing: EK; supervision: SK and PH. All authors contributed to the article and approved the submitted version.
